# Collodion in in-Growing Toe-Nail

**Published:** 1851-12

**Authors:** 


*Collodion in In-growing Toe-nail.*—[M. Meynier, in Bull. de Therap.] Press down the fleshy portion, and pour between it and the edge of the nail a small quantity of collodion; this soon solidifies, induces rapid cicatrization of the ulceration, and when the disease does not arise from an abnormal shape of the nail, procures a cure.

